# Effects of Freeze-Thaw Cycles on Phosphorus from Sediments in the Middle Reaches of the Yarlung Zangbo River

**DOI:** 10.3390/ijerph16193783

**Published:** 2019-10-08

**Authors:** Ning Liao, Lai Jiang, Jia Li, Linglei Zhang, Jing Zhang, Zeyu Zhang

**Affiliations:** 1 Institute of Ecology and Environment, State Key Laboratory of Hydraulics and Mountain River Engineering, College of Water Resource & Hydropower, Sichuan University, Chengdu 610065, China; liaoning16@hotmail.com (N.L.); zjing428@163.com (J.Z.); zzy4656@163.com (Z.Z.); 2 PowerChina Huadong Engineering Corporation Limited, Hangzhou 311122, China; jiang_l8@ecidi.com

**Keywords:** Yarlung Zangbo River, freeze-thaw cycle, sediment, phosphorus

## Abstract

The effect of the freeze-thaw process is an important factor in soil nutrient changes and erosion enhancement. Sediments in the middle reaches of the Yarlung Zangbo River are likely affected by the daily freeze-thaw cycles in winter. Examining the freeze-thaw effects of phosphorus from sediments in this area is of great significance for protecting the structure and safety of the ecosystem. The freeze-thaw process of sediments in the middle reaches of the Yarlung Zangbo River was simulated through laboratory experiments, and different phosphorus contents and particle states were synchronously detected and analyzed. The results show that freeze-thaw cycles can accelerate phosphorus migration and release in the sediments, and the total amount of phosphorus release increases by 12%. After being subjected to freeze-thaw cycles, the sediment particles were broken, and the competition between ions for adsorption sites reduced phosphorus adsorption onto the sediments from the middle reaches of the Yarlung Zangbo River. The organic matter on the sediment surface was also broken down, and the energy dispersive spectroscopy (EDS) results showed that the combined ions that were released competed for the adsorption sites on the particle surfaces, thereby promoting phosphorus release. Among the different forms of phosphorus, aluminum-bound phosphorus (Al-P) and iron-bound phosphorus (Fe-P) are the two most released phosphorus forms by the freeze-thaw process. Although the contents of Al-P and Fe-P only account for 2.41% of the total phosphorus content, both phosphorus forms are biologically available, and freeze-thaw cycles may increase the risk of nutrient loss. This research may provide information for the study of phosphorus in river ecosystems in areas subjected to freeze-thaw cycles.

## 1. Introduction

As one of the main source factors in river ecosystems, phosphorus plays an important role in maintaining the structure and function of river ecosystems [1,2]. Phosphorus can exist in many forms in soil. When the environment changes, some forms of phosphorus can be released from the soil into the ecological cycle, and the phosphorus in river sediments becomes the source and sink of the river ecosystem [3,4]. The freeze-thaw process strongly affects the release of soil phosphorus, therefore compromising the security and stability of ecosystems in areas subjected to freeze-thaw cycles [5,6]. The Yarlung Zangbo River originates from the Qinghai-Tibet Plateau, which is known as the “World Water Tower”, with an average altitude of more than 3700 m [7]. Affected by the plateau in this area, there is little cloud coverage over the ground, meaning that there is poor weakening of solar radiation during the day and poor reflection of the surface heat radiation at night, resulting in a large temperature difference between day and night [8]. In winter, freeze-thaw cycles occur daily along the banks of the middle reaches of the Yarlung Zangbo River [9]. However, research on the effect of freeze-thaw cycles on phosphorus from sediments in the middle reaches of the Yarlung Zangbo River is still lacking, which has become an urgent problem to be solved for protecting the river ecosystem of the Yarlung Zangbo River.

There are many endemic species in the river ecosystem of the Yarlung Zangbo River, and its ecological significance has a unique and important position in the world [10]. Influenced by the plateau environment, the river ecosystem of the Yarlung Zangbo River is simpler and more vulnerable than other plain rivers [7], causing the role of phosphorus in maintaining the stability of river ecosystems to be more prominent. The middle reaches of the Yarlung Zangbo River are in one of the most weathered areas in the world, and the primary weathered sediments are widely distributed here [11]. When sediment is frozen and thawed, these conditions may lead to a special response in terms of the migration and release of phosphorus in the sediments. There have been many reports on the effects of freeze-thaw cycles on phosphorus release in the past; these cycles change the functional structure and physical and chemical properties of soil, resulting in changes in the adsorption characteristics of phosphorus [12,13]. Sorption is one of the main mechanisms of phosphorus retention in soils, and phosphate adsorption occurs mainly on clay particles in soil, especially iron and aluminum oxides [14,15]. In addition, the organic matter bound to iron and aluminum complexes is widely present in various soil matrices [16]. The freeze-thaw process alters the binding states of iron, aluminum and organic matter, thus changing the capacity of soil to absorb phosphorus, but this change varies greatly because of the different freeze-thaw soil characteristics [6,13]. Wang et al. [5] and Riddle et al. [17] conducted similar freeze-thaw studies on wetland and cultivated land soil, respectively, and found that the ability to adsorb phosphorus increased and decreased, respectively. However, most researchers employ single soil types as the research object, such as wetlands [5,18] and cultivated land [17,19], while there are few studies [20,21] on phosphorus migration between soil and sediment types. Soil is usually composed of porous aggregates and has a long freeze-thaw period; frost heave can break up aggregates and interfere with the migration and release of some forms of phosphorus in soil [22]. The sediments in the middle reaches of the Yarlung Zangbo River are mostly loose grains [11], with short freeze-thaw cycles due to the diurnal temperature difference. The sediment properties and environment in this area are unique and are quite different from those of general soil.

There have been extensive and in-depth studies on the release of soil phosphorus under the action of freeze-thaw cycles. However, uncertainties remain, and the previous research results may not be applicable to the middle reaches of the Yarlung Zangbo River. In this paper, the surface sediments in the middle reaches of the Yarlung Zangbo River are selected for laboratory simulation experiments. The purpose is to explore the migration and release of phosphorus and the internal mechanism in sediments in the middle reaches of the Yarlung Zangbo River under freeze-thaw cycles from different forms of phosphorus and different sediment particle states. This research may provide a basis and reference for the prediction, protection and control of the Yarlung Zangbo River ecosystem.

## 2. Materials and Methods

### 2.1. Study Area and Sampling Sites

The Yarlung Zangbo River is located in the Tibet Autonomous Region of China, with a drainage area of 246,000 square kilometers [23]. The main cities and industries in Tibet are scattered around the river bank. The total length of the main stream is 2057 kilometers, which can be divided into upstream, middle and downstream sections [8]. The middle section is from Lhaze to Pai Town, with a total length of 1340 km, accounting for 65% of the total length of the river, with an average elevation of 3700 m. In winter, the temperature difference between day and night can reach 25 °C, leading to freezing and thawing cycles along the coast [10].

**Figure 1:**
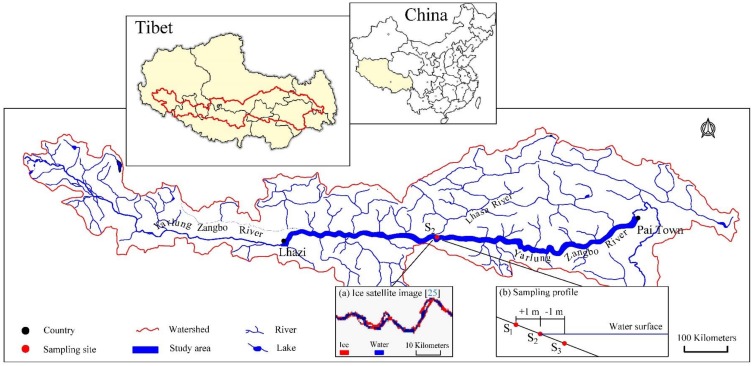
Study area and sampling sites.

The middle reaches of the Yarlung Zangbo River were selected as the study area (Figure 1). According to the investigation results of Chen et al. [24] on the phosphorus background value of sediments in the middle reaches of the Yarlung Zangbo River, the section with a relatively high phosphorus content was selected as the actual sampling section. According to the Yarlung Zangbo River ice satellite image [25] (Figure 1a), the specific location of the freeze-thaw area along the midstream section (S_2_, 29°20′6.49′′ N, 90°42′4.30′′ E) was determined, and samples were collected along the riverbank in September 2018. Considering the fluctuation in the water level and the sampling error, two sampling positions (S_1_ and S_3_, Figure 1b) were added along the axis of the section by ±1 m during sampling. The surface sediments were collected by the five-point sampling method at each position, and the microorganisms were dispatched at the sampling site.

### 2.2. Experimental Plan

After the sediment sample was transported back to the laboratory, it was first dried at room temperature (15 ± 1 °C) with an air dryer; this process lasted for 2 weeks. When the moisture content was less than 5%, sediment particles of different sizes were screened by 10 mesh and 240 mesh sieves, and the different forms of phosphorus at each particle size were analyzed. To ensure that the experimental results were representative and indicative of the particle state, the sediment particle size with the highest phosphorus content was selected as the experimental sample.

The experiments were carried out in four plexiglass columns with a diameter of 12 cm and a height of 14 cm; two experiments constituted the freeze-thaw cycle group, and two experiments constituted the constant-temperature control group. According to the actual water-soil ratio in the freeze-thaw area of the river reach, the water-soil ratio in the device was set to 5:1, and a 2 cm thick sediment layer was laid on the bottom of the columns. Distilled water was used for the overlying water. During winter, ice appeared in the middle reaches of the Yarlung Zangbo River at approximately 2 o’clock and began to melt at 11 o’clock. The freezing time lasted for 9 h, and the temperature along the shore ranged from −14 °C to 12 °C. Taking these data as the experimental environmental control conditions, the freeze-thaw cycle group was subjected to freeze treatment at -14 °C for 9 h and then placed in a constant-temperature aseptic incubator at 12 °C for 15 h. One freeze-thaw cycle lasted 1 day, and cycle experiments were performed for a total of 10 days. The constant-temperature control group was subjected to a fixed temperature of 12 °C in an incubator. During the experiment, some surface sediments of each columns were sampled before freezing every day for analysis of various indicators.

### 2.3. Test Methods

The chemical sequential extraction method [26] was used to gradually extract soluble phosphorus (SP), aluminum-bound phosphorus (Al-P), iron-bound phosphorus (Fe-P), calcium-bound phosphorus (Ca-P), occluded phosphorus (Oc-P) and organic phosphorus (OP). NH_4_Cl, NH_4_F, NaOH, H_2_SO_4_ and other reagents were used. Oscillation, centrifugation, heating, and other operations were conducted in the overall extraction procedure. Molybdenum antimony anti-spectrophotometry was carried out to determine the concentration of phosphorus via UV spectrophotometry (TU-1950, PERSEE, Beijing, China).

To examine the intrinsic mechanism of the release and migration of phosphorus, the organic matter content, specific surface area, particle size and elemental composition of the sediment samples were determined. The organic matter content was determined by the potassium dichromate-sulfuric acid digestion method [27]. The specific surface area of the sediment particles was determined by nitrogen adsorption using an automatic specific surface area and porosity analyzer. The particle size and elemental composition were obtained by scanning electron microscopy and energy dispersive spectroscopy (SEM-EDS) (REGULUS8100 SEM, HITACHI, Tokyo, Japan; ASAP2460 BET, Micromeritics Instrument, Atlanta, GA, USA).

### 2.4. Statistical Analysis

Three samples were taken from each column at each time of sampling and determined. The data are presented in the figures as the dry mass average concentration of the samples and reported as the means ± SD. One-way analysis of variance (one-way ANOVA) and Duncan’s multiple range test (DMRT) were performed using SPSS 18.0 (IBM Inc., United States) to test for significant differences among the groups. Boltzmann curves were used to fit the regularities of the average concentrations of the various forms of phosphorus in the sediments (R^2^ > 0.5). Pearson correlation analysis of the measured parameters was conducted.

## 3. Results

### 3.1. Particle Size and Phosphorus Occurrence Characteristics

According to the sediment classification method [28], the sediment particle sizes obtained by screening were divided into fine particles (Φ < 0.063, where Φ is the particle size, mm), medium-sized particles (0.063 < Φ < 2.0) and coarse particles (2.0 < Φ). The results showed that the mass fraction of the medium-sized particle in the sediments was the largest (Table 1), and the average value was 52.47%, which is 3.6 and 1.6 times higher than the fine and coarse particle mass fractions, respectively. The standard deviation of the mass fraction of the medium-sized particle was 0.9%, that of the fine particle was 1.3%, and that of the crude particle was 2.0%. These results indicated that the sediments in the middle reaches of the Yarlung Zangbo River were dominated by medium-sized particles and were distributed evenly at all points.

**Figure 2:**
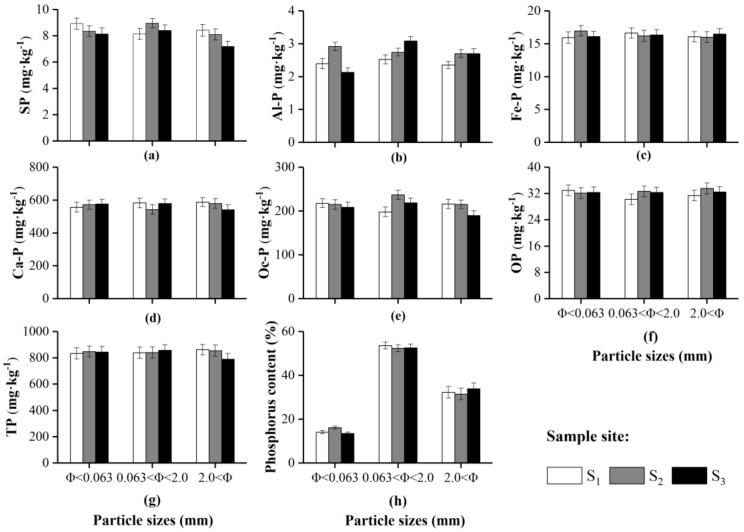
Background values of various forms of phosphorus from sediments with different particle sizes at three sampling sites. Data are displayed as means ± standard deviations.

Figure 2 shows the various forms of phosphorus content in the sediments at different particle sizes, and the total phosphorus content in the sediments ranged from 847.54 to 862.41 mg·kg^−1^. The inorganic phosphorus content ranged from 815.38 to 823.73 mg·kg^−1^, and inorganic phosphorus was the main form of phosphorus in the sediments in the middle reaches of the Yarlung Zangbo River. There was no significant difference in the contents of the different forms of phosphorus among the different particle sizes. The content of SP was approximately 1.02%, the Al-P content was approximately 0.33%, the Fe-P content was approximately 2.08%, the Ca-P content was approximately 67.79%, the Oc-P content was approximately 25.06% and the OP content was approximately 3.72%, which indicates that the differences in phosphorus contents among the different particle sizes was determined by the mass fraction. Combined with the mass fractions of the sediment particle sizes and layers, the results showed that the phosphorus contents in the medium-sized clastic sediments were the highest.

### 3.2. Migration and Release of Phosphorus

**Figure 3:**
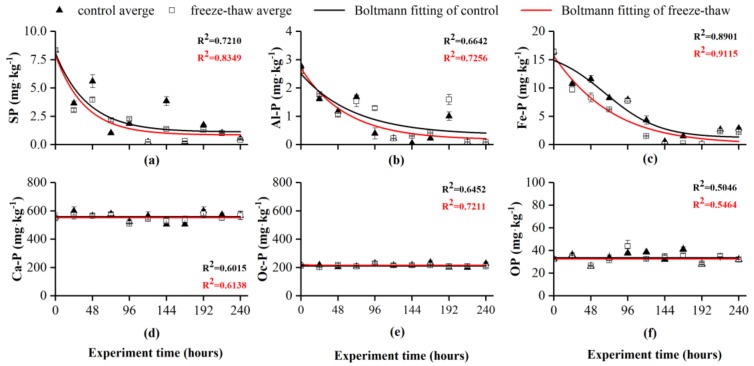
Comparison of the phosphorus concentration in sediments between the control group and the freeze-thaw group during the experiment. Data are displayed as means ± standard deviations.

Figure 3 shows a comparison of the phosphorus concentration in sediments between the control group and freeze-thaw group during the experiment. The concentrations of SP, Al-P and Fe-P in the sediments decreased rapidly within 144 hours. The SP concentration in the two groups decreased rapidly after stabilization, and the stable concentration in the freeze-thaw group was slightly lower than that in the control group. In the freeze-thaw group, the concentration of Al-P in the sediments decreased faster within 144 hours than that in the constant-temperature group, and the concentration of the freeze-thaw and control groups after stabilization reached 0.218 mg·kg^−1^ and 0.415 mg·kg^−1^, respectively. The Fe-P concentration in the sediment was affected by freezing-thawing within 72 hours, and the rate of decline was 13%~28% higher than that of the constant-temperature group. The Fe-P concentration in the two groups stabilized at 144 hours, and the stable concentrations of the freeze-thaw and control groups were 1.965 mg·kg^−1^ and 2.183 mg·kg^−1^, respectively; the release of phosphorus in the freeze-thaw group was greater than that in the control group. Boltzmann curve fitting showed that the concentrations of Ca-P and Oc-P in the sediments remained unchanged (R^2^ > 0.6), showing a stable state. The concentration of OP fluctuated slightly during the experiment, and the fitting results showed that the concentration of OP remained unchanged (R^2^ > 0.5).

### 3.3. Particle Size Change

**Figure 4:**
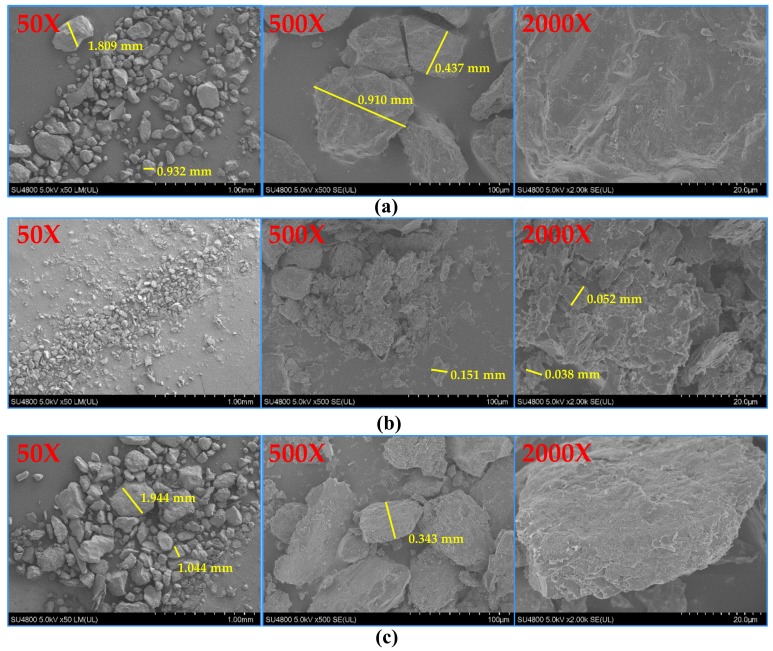
Measurements (yellow line) of the sediment particles before and after the experiment: (**a**) before the experiment, (**b**) after 240 hours of freeze-thaw cycles, (**c**) control group, with 50x, 500x, and 2000x amplification.

Figure 4 shows the particle size changes in the sediment samples before and after the experiment. Before the experiment, the medium-sized clastic sediment particles were more uniform than after the experiment, and the scanning results magnified 2000 times (2000X) showed that the surface of the particles was smooth. After 240 hours of freeze-thaw cycles, there were a large number of particles smaller than 0.063 mm in the sediment samples. The measurements showed that the particle size of some particles reached 0.038 mm after crushing, and amplification at 2000X showed that the surface of the particles was severely fragmented. In the control group, no particle fragmentation was observed after the experiment, but the particle surface was not as smooth as that of the sample before the experiment, and the particle surface was rougher.

Table 2 shows the change in the specific surface area of the sediment particles before and after the experiment. The results showed that the specific surface area of the particles before the freeze-thaw cycle was 1.3673 m^2^·g^−1^, and the t-plot micropore area was 0.5164 m^2^·g^−1^, indicating that the sediments in the middle reaches of the Yarlung Zangbo River are nonporous particles. After the freeze-thaw cycles, the specific surface area of the sediment particles was 2.623 m^2^·g^−1^, which represented an increase of 92%, and the t-plot micropore area was 0.6028 m^2^·g^−1^, which was an increase of 17%. These results prove that freeze-thaw cycles can cause sediment particle breakage. In the control group, after the freeze-thaw cycles, the specific surface area of the sediment particles increased by 0.3145 m^2^·g^−1^, which was an increase of 23%, and the micropore area was 0.0074 m^2^·g^−1^, which was a decrease of 99%.

### 3.4. Particle Composition Change

**Figure 5:**
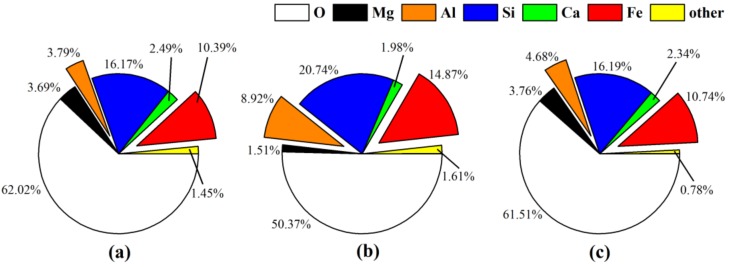
Elemental percentages from sediment particles before and after the experiment: (**a**) before the experiment, (**b**) after 240 hours of a freeze-thaw cycle, and (**c**) control group.

Figure 5 shows the changes in the elemental composition of the sediment grains before and after the experiment. The results showed that the atomic content of the oxygen atom (O) is dominant in the sediment grains before the experiment, accounting for 62.02% of the total atomic content, and the atomic contents of aluminum (Al), silicon (Si) and iron (Fe) were 3.79%, 9.57% and 10.39%, respectively. After the freeze-thaw cycle, the atomic content ratio of O and magnesium (Mg) decreased by 11.65% and 2.18%, respectively. The atomic contents of Al, Si and Fe increased to 8.92%, 20.74% and 14.87%, respectively, after the freeze-thaw cycle. No other changes were observed in the detected elements. In the particles of the control group, after the freeze-thaw cycle, the proportion of the O atom content decreased by 0.51%, while the atomic contents of Al and Fe increased 0.89% and 0.35%, respectively, but the increase rate was lower than that in the particles of the freeze-thaw group.

**Figure 6:**
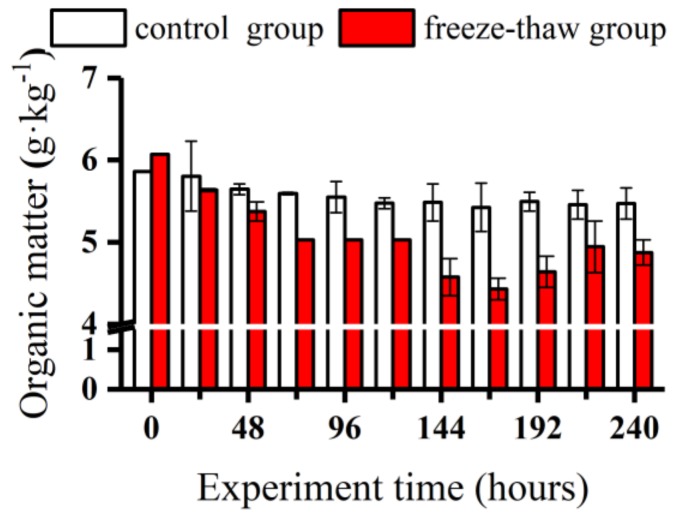
Organic matter content from sediments of the control and freeze-thaw groups during the experiment. Data are displayed as means ± standard deviations.

Figure 6 shows the changes in the organic matter content in the sediments of each group during the experiment. The initial organic matter content in the sediments in the middle reaches of the Yarlung Zangbo River was 6.06 g·kg^−1^. As the experiment progressed, the organic matter content in the sediments of the control group decreased slightly, with values ranging from 5.42 to 5.81 g·kg^−1^, while that in the sediments of the freeze-thaw group decreased gradually and stabilized at 4.87 g·kg^−1^. A correlation analysis of the contents of organic matter and the various forms of phosphorus in the sediments showed that there was a significant positive correlation between the organic matter and Fe-P contents (Table 3) (0.94, *p* < 0.01), and there was a positive correlation between the organic matter content and the SP and Al-P contents (0.76 and 0.64, respectively; *p* < 0.05).

## 4. Discussion

### 4.1. Influencing Mechanism of the Freeze-Thaw Cycle

The simulation results showed that the sediment particles were broken in the middle reaches of the Yarlung Zangbo River during the freeze-thaw cycle. With the continuous freeze-thaw cycle, the specific surface area of the particles increases, which also proves that the particles are broken. The cause of this phenomenon may be related to the frost heave force described by Chai et al. [29], because when freeze-thaw cycles occur, the freezing process results in ice crystals, which produce a frost heave force. The ice crystals melt and shrink with increasing temperature, and the repeated process of ice crystal expansion and shrinkage deteriorates the bonding state of large particles, thus splitting these large particles into small particles. Before the experiment, the specific surface area of the sediment particles was 1.3673 m^2^·g^-1^, which was much smaller than the specific surface area of generally occurring porous particles (greater than 10 m^2^·g^−1^) [30], indicating that the sediments in the middle reaches of the Yarlung Zangbo River were nonporous particles. The SEM-EDS analysis results showed that the samples contained Fe, Al and Mg, indicating that certain clay minerals were present in the sediments in the middle reaches of the Yarlung Zangbo River, and the clay minerals were only adsorbed onto the surface of the sediment particles. Clay minerals are major adsorbents in soil, which can adsorb large amounts of organic matter and release these organic substances into the water environment when conditions change [31]. The atomic ratio of Si/O is 1/4, which is much lower than that of Si/O in clay minerals (approximately 1/3 or 2/5) [32], and no other nonmetallic elements except Si and O were detected, indicating that the organic matter on clay minerals is present in a certain form.

After 240 hours of the freeze-thaw cycle experiment, the organic matter content in the sediments in the middle reaches of the Yarlung Zangbo River decreased by 19.6%, indicating that the organic matter adhering to the surface of sediment particles changes chemically after it is crushed by freeze-thaw cycle processes. The increase in Al, Fe and Si atoms in the sediment particles means that these elements are released from the organic matter under the freeze-thaw action, and the increase in oxygen consumption during the release process leads to a decrease in the O atom content. This result confirms the hypothesis presented by Leanne et al. [33] that freeze-thaw cycles change the organic matter morphology of soil, and among the present elements, Fe and Al mineral elements react most intensely during the freeze-thaw process; these reactions may consume oxygen and release carbon dioxide.

The results show that the freeze-thaw process can promote the release of Al-P and Fe-P from sediments in the middle reaches of the Yarlung Zangbo River. An analysis of the adsorption mechanism indicates that, on the one hand, the SEM results showed that sediment particles became fragmented during the freeze-thaw cycles, the particle size became smaller, and the specific surface area increased by 92%, which may reduce the agglomeration of sediment particles and increase the surface adsorption point [34], which enhances the adsorption capacity of sediments. On the other hand, the fragmentation of sediment particles deteriorates the binding state of organic matter with Fe and Al complexes and leads to the release of Fe and Al compounds into the interstitial solution of the sediments. These re-released ions compete with phosphate for the adsorption sites on particles [35], even replacing the phosphate originally adsorbed onto the surface of the sediment particles, resulting in a decrease in the phosphorus adsorption capacity of sediments. After the freeze-thaw treatment, the phosphorus adsorption capacity of sediments may be reduced more than enhanced, resulting in the release of these two forms of phosphorus.

The results also indicated SP release in the freeze-thaw and control groups, and the release rate was consistent. As SP is soluble in water and the overlying water used in the experiment is distilled water, there is a difference in the phosphorus concentration between the two interfaces, which leads to the migration and release of SP into the overlying water along the concentration gradient. The contents of Ca-P and Oc-P in the sediments are the dominant forms of phosphorus, accounting for 92.8% of the total phosphorus, which confirms that self-generated phosphorus is the main phosphorus form in the middle reaches of the Yarlung Zangbo River on the basis of a report by Cheng et al. [24]. However, the contents of these two phosphorus forms do not change with freeze-thaw cycles; that is, Ca-P and Oc-P are stable and insoluble in water and are not easily affected by environmental changes, which is consistent with the prevailing conclusions [36]. In conclusion, the adsorption capacity of the sediments in the middle reaches of the Yarlung Zangbo River after freeze-thaw cycles is reduced, which mainly promotes the release of Al-P and Fe-P.

### 4.2. Comparisons between the Yarlung Zangbo River and Other Regions

Freeze-thaw cycles affect the concentration of phosphorus in sediments mainly by changing the adsorption of phosphorus in sediments, but previous conclusions on the effect of freeze-thaw cycles on phosphorus adsorption in soils are different. Four different soil properties or freeze-thaw conditions (Table 4) were compared to explore the mechanism by which the desorption capacity of sediments in the middle reaches of the Yarlung Zangbo River may be greater than the adsorption capacity after freeze-thaw cycles. Different freeze-thaw experimental studies indicated that the ability of soil to absorb phosphorus is reduced [37,38], enhanced [39] or unchanged [19] by freeze-thaw cycles. Through comparative analysis, on the one hand, the sediments in the middle reaches of the Yarlung Zangbo River are porous particles with an organic matter content of only 6.06 g·kg^−1^, which is much lower than that of pure clays. Particles with a particle size of 0.063 < Φ < 2 accounted for more than 50% of the total particles, indicating that the sediments in this study can be classified as sandy soil and that the sediment particles themselves have low adsorption capacity. Zheng et al. [11] also revealed similar laws, showing that in the past 8600 years, the sediments in the middle reaches of the Yarlung Zangbo River have been affected by the unique climate, such as cold and dry, strong winds, and the sediments are sandy immature soils. Although the freeze-thaw cycle enhances the adsorption of sediment particles after they are broken, due to the nature of the sediment, the desorption capacity due to the competition between ions and phosphate for adsorption sites still exceeds the adsorption capacity. Zhao et al. [37] studied the soil of Keerqin sandy land, and the freezing and thawing conditions were set to freeze for 10 days at −12 °C; they found that the freeze-thaw cycle reduced the soil’s ability to absorb phosphorus. However, their freeze-thaw conditions are different from those in this study, and the effect of phosphorus release is the same when the soil is the same (sandy soil). In the study by Özgül [39], lacustrine residual sediments were used for freeze-thaw experiments, and the lacustrine residual sediments were mainly composed of large amounts of clay, humus and gravel, which were more capable than sandy soil of adsorbing phosphorus. Even if ionic competition resulted in desorption, adsorption is still stronger than desorption, and this conclusion was confirmed by Wang et al. [5].

On the other hand, each experimental freeze-thaw study is dependent on the characteristics of the study area; the experimental conditions for freezing and thawing are also different and can be divided into short-cycle and long-cycle freeze-thaw conditions [40]. The sediments in the middle reaches of the Yarlung Zangbo River differ greatly from the brown forest soil in Northeast China. Under short-term freeze-thaw cycles, both types of studies have the common characteristics of promoting soil phosphorus release. This is because short-cycle freeze-thaw conditions not only break down soil particles but also freeze water rapidly before the particles can collapse and precipitate, which loosens the soil structure compared with soil subjected to long-term freeze-thaw cycles; in addition short-cycle freeze-thaw conditions are more conducive to the lateral migration of substances, and the adsorption capacity is further reduced. Although the experimental soils such as those used in the studies by Qian et al. [38] and Peltovuori et al. [19] were all clays, the effects of different periods on the phosphorus adsorption capacity of the soils were also different. In summary, the sediments in the middle reaches of the Yarlung Zangbo River were subjected to freeze-thaw cycles, and the adsorption properties of the sediment, the desorption ability due to ion competition and the short-term freeze-thaw cycle reduced the phosphorus adsorption capacity of sediments more than they strengthened it; in addition, the release of Al-P and Fe-P was greater after final equilibrium was reached.

**Figure 7:**
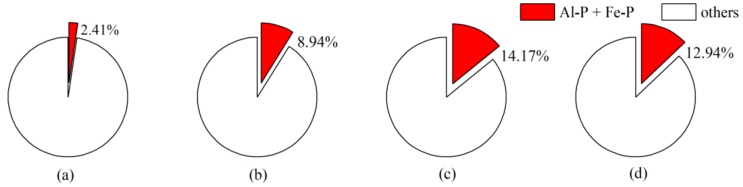
Proportion of the Al-P and Fe-P contents in sediments from different freeze-thaw areas in China: (**a**) the Yarlung Zangbo River, (**b**) the Liaohe River [41], (**c**) the Songhua River [42], and (**d**) the Haihe River [43].

The freeze-thaw treatment of the sediments in the middle reaches of the Yarlung Zangbo River mainly promoted the release of Al-P and Fe-P, which are important potential bioavailable phosphorus sources in river sediments [26]. To compare the effects of phosphorus from sediments in the middle reaches of the Yarlung Zangbo River subjected to freeze-thaw cycles on river basin ecosystem, the results of screened rivers in other freeze-thaw regions of China were compared (Figure 7); the sediments of these rivers were stimulated to release Fe-P and Al-P after freeze-thaw cycles, and these data are available in the literature [41,42,43]. The contents of Al-P and Fe-P in the sediments in the middle reaches of the Yarlung Zangbo River are only 2.41%, which is only approximately 1/6~1/4 of those in the sediments of the other three rivers, which may be closely related to the surrounding external inflow.

The middle reaches of the Yarlung Zangbo River have little vegetation, and the rock mass and soil are exposed all year [44]. Although weathered soil particles can enter rivers as external sources, they lack organic material, and primary phosphorus is mainly calcium-bound phosphorus, which makes it difficult for natural external inflow to increase the ratio of these two forms of phosphorus. The proportion of these two forms of phosphorus also increases with an increase in artificial external inflow [45]. According to the 2018 Tibetan Statistical Yearbook [46], the population density in Tibet is only 2.55 persons·km^−2^, which is much lower than that in the other three river regions, and the population growth rate is 1.06%, which implies that the human activities in the Yarlung Zangbo River region will not increase significantly for a long time. Although the contents of Al-P and Fe-P in the sediments are low, the total effect of phosphorus on the sediments is limited when the freeze-thaw process promotes the release of these two forms of phosphorus. However, the lack of external inflow supplementation may cause the freeze-thaw cycle to increase the risk of sediment phosphorus loss in the middle reaches of the Yarlung Zangbo River, and the risk is much greater in this river than in the other three rivers. As the Yarlung Zangbo River has a unique ecological status in the downstream East Asian region and in the global environment, any small fluctuation in these two forms of phosphorus may result in irreversible effects, which cause great uncertainty in the ecosystem; this issue requires more attention in the Yarlung Zangbo River than in the rivers of the other freeze-thaw regions.

## 5. Conclusions

In this paper, the effects of freeze-thaw cycles on phosphorus from sediments were monitored by controlling the temperature, soil-water ratio and cycle time in the laboratory under winter conditions reflective of the middle reaches of the Yarlung Zangbo River. The conclusions are as follows:

(1) The sediments in the middle reaches of the Yarlung Zangbo River can be divided into fine, medium-sized and coarse particles. The total phosphorus contents in the medium-sized particles were the highest among the different particle sizes, accounting for 52.47% of the total mineral content.
(2) Freeze-thaw cycles cause breakage of sediment particles, which increases the specific surface area and deteriorates the organic matter at the same time. The released ions compete for the adsorption sites on the surface of the sediment particles to promote the release of phosphorus, and the release increases by 12%, which mainly promotes the release of Al-P and Fe-P.
(3) The freeze-thaw process affects the phosphorus adsorption capacity of sediments, and the overall magnitude of the influence is related to the characteristics of the sediment particles and the freeze-thaw cycle. The sediments in the middle reaches of the Yarlung Zangbo River are immature sandy soils, and freeze-thaw cycles decrease the phosphorus adsorption capacity of such sediments.
(4) The Al-P and Fe-P contents account for only 2.41% of the total sediment phosphorus content, and freeze-thaw cycles promote the release of these two forms of phosphorus in sediments with a limited overall impact but may accelerate the loss of bioavailable phosphorus in sediments, which needs to be investigated further.

## Figures and Tables

**Table 1:** Mass fraction (%) of the sediment particle size hierarchy at the different sample sites.

Sample Site	Particle Size (mm)		
	Φ < 0.063	0.063 < Φ < 2.0	2.0 < Φ
S_1_	14.27	53.45	32.28
S_2_	16.24	52.69	31.07
S_3_	13.01	51.26	35.73
Average	14.51	52.47	33.03

**Table 2:** Changes in the specific surface area of the sediment particles before and after the experiment.

**Area**	**Single-Point Surface Area**	**BET Surface Area**	**Langmuir Surface Area**
Original (m^2^·g^−1^)	1.3296	1.3673	3.8519
Freeze-thaw (m^2^·g^−1^)	2.5593	2.623	8.3803
Range (%)	92	92	118
Control (m^2^·g^−1^)	1.5737	1.6818	4.5745
Range (%)	18	23	19
**Area**	**T-plot micropore area**	**T-plot external surface area**	
Original (m^2^·g^−1^)	0.5164	0.8508	
Freeze-thaw (m^2^·g^−1^)	0.6028	2.0202	
Range (%)	17	137	
Control (m^2^·g^−1^)	0.0074	0.9251	
Range (%)	−99	9	

**Table 3:** Pearson’s correlations among different forms of phosphorus concentrations and organic matter concentration.

Phosphorus	SP	Al-P	Fe-P	Ca-P	Oc-P	OP
Control	0.31	0.41	0.62 *	0.19	−0.37	−0.17
Freeze-thaw	0.76 *	0.64 *	0.94 **	0.19	−0.18	−0.13

Note: ** indicates *p* < 0.01, * indicates *p* < 0.05.

**Table 4:** Phosphorus adsorption conclusions from different freeze-thaw studies.

**Similar Studies**	**Yarlung Zangbo River**	**Keerqin Sandy Land [37]**	**Northeastern China [38]**
Research soil	nonporous sediment	sandy soil	Brown forest soil
Experimental treatments	−14 °C (9 h)~12 °C (15 h), 10 cycles	freezing at −12 °C for 10 days	−10 °C~7 °C, each cycle lasts 12 h, 6 cycles
Adsorption change	reduced	reduced	reduced
**Similar studies**	**Turkish** **highland [39]**		**Finland [19]**
Research soil	marn, lacustrine residual sediment, etc.		cultivated mineral soils
Experimental treatments	freezing at −10 °C for 30 days, thawing at 2.5 °C, 3 cycles, etc.		freezing at −18 °C for 21 months, thawing at 5 °C for 1 weeks
Adsorption change	enhanced		unchanged
